# Reduced Parallel Gene Expression Evolution With Increasing Genetic Divergence—A Hallmark of Polygenic Adaptation

**DOI:** 10.1111/mec.17803

**Published:** 2025-05-16

**Authors:** Dagny A. V. Thorhölludottir, Sheng‐Kai Hsu, Neda Barghi, François Mallard, Viola Nolte, Christian Schlötterer

**Affiliations:** ^1^ Institut für Populationsgenetik, Vetmeduni Vienna Vienna Austria; ^2^ Vienna Graduate School of Population Genetics Vienna Austria

**Keywords:** convergent evolution, divergence, gene expression evolution, parallel evolution, regulatory evolution

## Abstract

Parallel evolution, the repeated evolution of similar traits in independent lineages, is a topic of considerable interest in evolutionary biology. Although previous studies have focused on the parallelism of phenotypic traits and their underlying genetic basis, the extent of parallelism at the level of gene expression across different levels of genetic divergence is not yet fully understood. This study investigates the evolution of gene expression in replicate *Drosophila* populations exposed to the same novel environment at three divergence levels: within a population, between populations and between species. We show that adaptive gene expression changes are more heterogeneous with increasing genetic divergence between the compared groups. This finding suggests that the adaptive architecture—comprising factors such as allele frequencies and the effect size of contributing loci—becomes more distinct with increasing divergence. As a result, this leads to a reduction in parallel gene expression evolution. This result implies that redundancy is a crucial factor in both genetic selection responses and gene expression evolution. Hence, our findings are consistent with the omnigenic model, which posits that selection acts on higher‐order phenotypes. This work contributes to our understanding of phenotypic evolution and the complex interplay between genomic and molecular responses.

## 1 Introduction

The term ‘parallel evolution’ is used to describe the phenomenon of repeated phenotypic changes in independent groups in response to environmental stressors. Some researchers differentiate between parallel evolution, which occurs when the genetic basis of adaptation is shared, and convergent evolution, which occurs when genetically diverged groups have distinct genetic bases of adaptation (Bolnick et al. 2018). The interest of evolutionary biologists in parallel evolution lies not only in the ability to distinguish between stochastic and directed changes, but also in its potential for studying the predictability of evolution (Hartke et al. 2021; Jacobs et al. 2020; Nosil et al. 2018; Poore et al. 2023; Thorpe 2017; Walden et al. 2020; Zhao et al. 2015).

Extending the parallelism of traits to their genetic basis is not straightforward. In the case of traits with a simple genetic basis, the expectations of high parallelism (Fisher 1930; Orr 1998, 2005) can be directly transferred to genetic changes. Conversely, traits with a complex genetic basis, which encompasses the majority of ecologically relevant traits (Barghi et al. 2020; Savolainen et al. 2013), may not experience a parallel genetic response. The occurrence of parallel phenotypic changes in complex traits despite the use of different combinations of genes can be attributed to genetic redundancy (Barghi et al. 2019; Bolnick et al. 2018; Goldstein and Holsinger 1992; Láruson et al. 2020). This phenomenon occurs when more genetic variation is segregating in a population than needed to achieve the desired phenotypic change. The extent of parallelism for the genetic basis of polygenic traits is contingent upon the distance to the trait optimum and the adaptive architecture, which encompasses the distribution of effect sizes, allele frequencies, the number of contributing loci and pleiotropy (Barghi et al. 2020). In other words, it is the realised redundancy which determines the extent of parallelism. It is therefore not surprising that some studies have repeatedly identified the same gene or even the same variant in multiple lineages (Colosimo et al. 2005; Horton et al. 2021), whereas others have observed heterogeneous responses (Barghi et al. 2019; Poore et al. 2023).

An interesting alternative to studying the parallel response of different genes contributing to the same trait is to compare the parallelism of a given gene across different levels of genetic divergence. Some elements of the adaptive architecture, particularly allele frequencies and the effect size of the contributing loci, are expected to become more distinct with increasing divergence. Hence, less parallelism is expected for genes contributing to complex traits. Empirical evidence for this prediction comes from the diminishing genomic parallelism with increasing divergence in *Arabidopsis* (Bohutínská et al. 2021) and other species (Bohutínská and Peichel 2023). As a consequence, parallelism at the genomic level becomes increasingly complex with greater divergence, making it difficult to predict or infer phenotypic adaptation from (non)‐parallel evolution.

Gene expression is a molecular phenotype, which can be easily measured for many genes in a single experiment. In contrast to genomic variation, which typically focuses on single SNPs, gene expression integrates multiple regulatory information from *cis*‐ and *trans*‐acting factors (Cheung et al. 2010; Gilad et al. 2008; Morley et al. 2004; Price et al. 2011). Hence, gene expression can be considered a quantitative trait (Albert and Kruglyak 2015; Boyle et al. 2017; Jansen and Nap 2001) and it is conceivable that differences in the adaptive architecture between species are levelled out, resulting in a parallel gene expression change with limited effects of genetic divergence. On the other hand, it is not clear how redundancy on the gene expression level translates into non‐parallel gene expression changes across genetically divergent groups.

**FIGURE 1:**
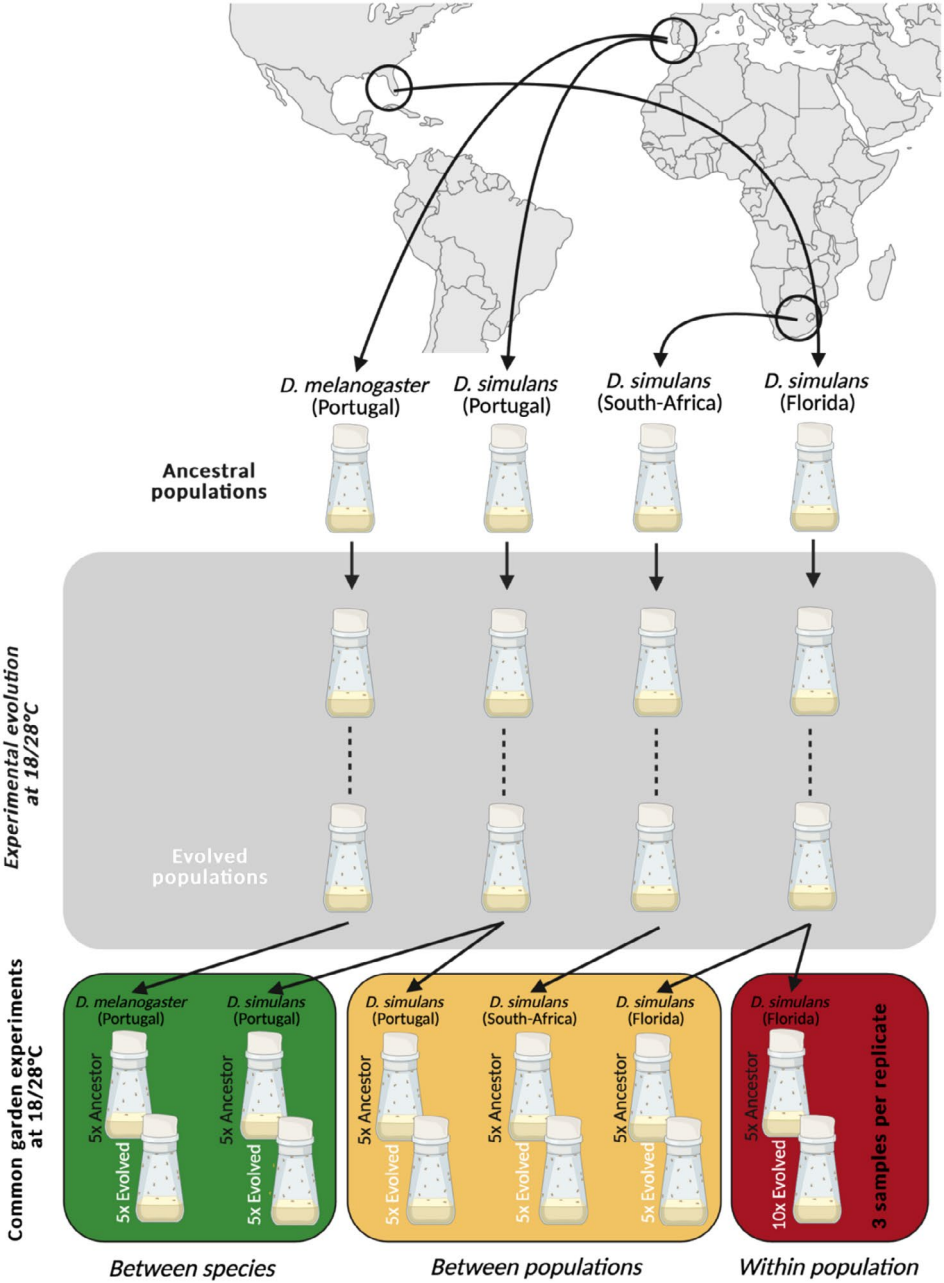
Experimental design. Experimental evolution of populations with different levels of genetic divergence in the same novel selection regime for > 85 generations. Each population is maintained in 5 or 10 replicates. Three common garden experiments (bottom panel) were performed to measure the evolved gene expression changes, one for each divergence level. Red for the lowest divergence level (within population). Yellow for the middle divergence level (between populations) and the highest divergence level in green (between species). Created with BioRender.

Here, we address the question of parallel gene expression evolution across genetically diverged groups by quantifying the evolution of gene expression in replicate populations exposed to the same novel environment at three divergence levels in *Drosophila* (within a population, between populations and between species) (Figure 1). We show that adaptive gene expression changes are increasingly dissimilar with increasing divergence between the compared groups. Consistent with the proposed increase in parallelism at higher hierarchical levels (Lai et al. 2023), we also notice more parallel responses after grouping genes by GO category.

## 2 Results

We investigated gene expression across varying levels of evolutionary divergence in three common garden experiments: (1) between 10 independently evolved replicates derived from the same *D. simulans* population, (2) between three geographically distinct *D. simulans* populations (Florida, Portugal, South Africa) and (3) between *D. simulans* and *D. melanogaster* populations from Portugal (Figure 1). Due to logistical constraints, separate common gardens were used for each divergence level, following the same protocol. For all experiments, evolved replicates were compared to ancestral populations under controlled conditions, with transcriptional profiles measured from whole body males after standardising density and environmental conditions for two generations to minimise transgenerational effect.

### 2.1 Adaptation of Evolved Population at All Divergence Levels

All evolved populations increased in fitness relative to their ancestors, which documents the successful adaptation to the new laboratory environment (Barghi et al. 2019; Mallard et al. 2018; Tobler et al. 2015). Since we did not measure fitness for all populations at the same time, we cannot make a statement about the convergence of fitness in all evolved populations. We also caution that measures of fitness, such as fecundity or egg to adult viability, are only measuring fitness components and therefore some heterogeneity may be detected for fitness components, even when populations are fully adapted and have reached the same trait optimum (Christodoulaki et al. 2022). Given these uncertainties about the interpretation of potential differences in fitness measurements, we did not consider this an important factor and assumed that the number of generations has been sufficiently large (> 85 generations, Table 1) that the populations have approached the trait optima of the new environment.

**TABLE 1:** Overview of the three common gardens for each of the divergence levels.

Common garden experiment	Species	Population	Generation	Replicates	Sub replicates
Within population	*D. simulans*	Florida	103	10	3
Between populations	*D. simulans*	Portugal	161	5	0
	*D. simulans*	Florida	131	5	0
	*D. simulans*	South‐Africa	85	5	0
Between species	*D. melanogaster*	Portugal	155	5	0
	*D. simulans*	Portugal	133	5	0

### 2.2 Gene Expression Changes After Adaptation to a Hot Laboratory Environment Indicate the Evolution of Various Biological Processes

Gene expression changed in all evolved populations (Figure S2). Between 366 and 2251 genes showed significant gene expression changes. The data revealed that genes with significantly different expression levels (FDR < 0.05) were enriched for 20–108 Gene Ontology (GO) terms (Supporting Information), across the experiments. This significant enrichment indicates an adaptive response for gene expression intensity, as random changes in expression levels across the transcriptome would be unlikely to result in significant enrichment. The observed functional implications of enriched GO categories associated with metabolism, neuronal biology and fatty acid synthesis (Supporting Information) have already been noticed previously and are discussed there (Hsu, Belmouaden, et al. 2020; Jakšić et al. 2020; Lai and Schlötterer 2022; Mallard et al. 2018, 2020; Thorhölludottir et al. 2023).

### 2.3 The Global Adaptive Response in Gene Expression Is Shared Across Different Divergence Levels

**FIGURE 2:**
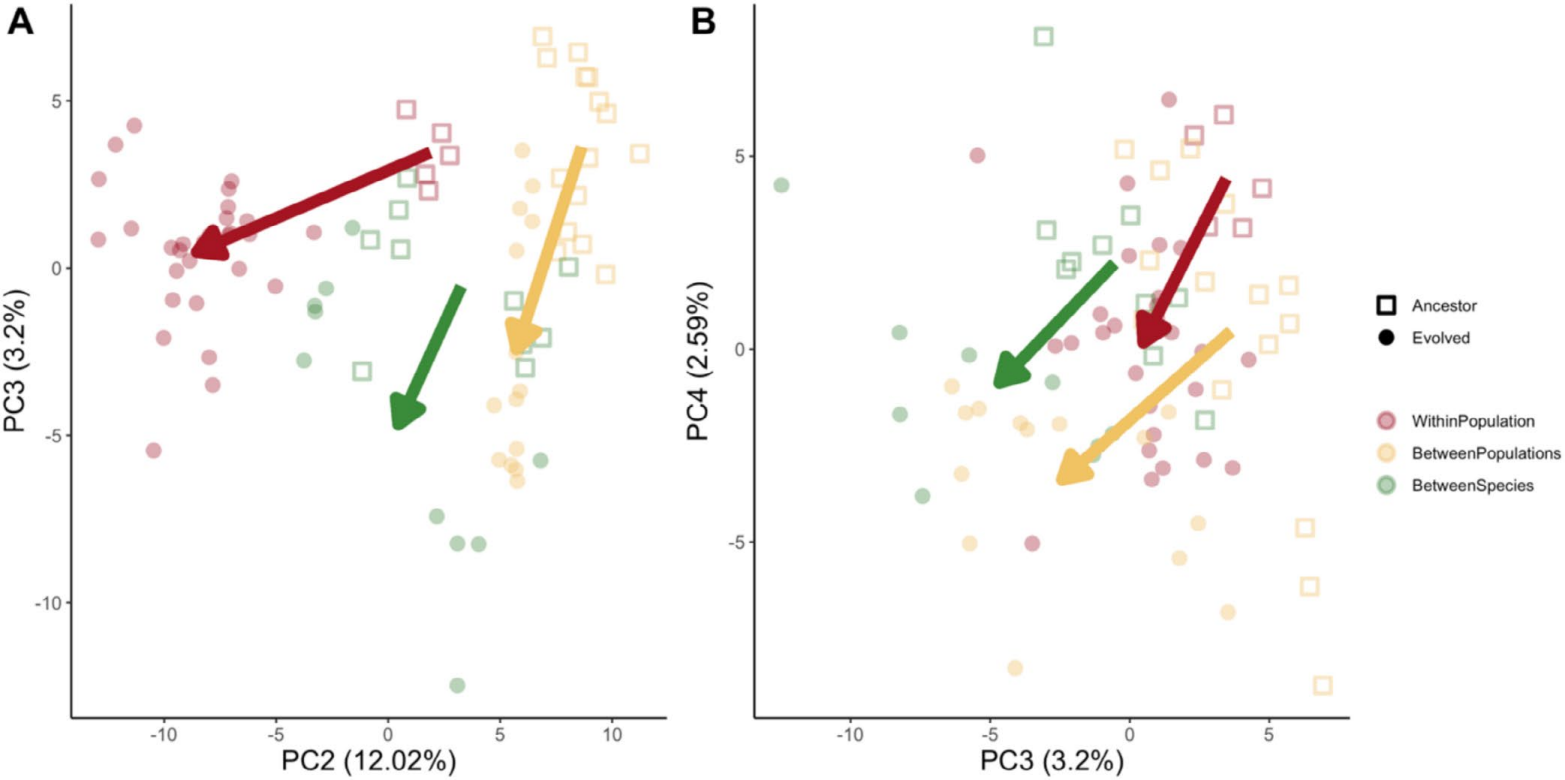
Parallel evolutionary response of the entire transcriptome during experimental evolution for three different levels of divergence. We used principal component analysis to determine the parallel response of the transcriptome to the same stressor on three different levels of divergence. The axes show PC2, PC3 and PC4, which are separating ancestors (open squares) from evolved samples (filled circles). PC1 separated the species and explained 50.05% of the variation in the data (Figure S1). Arrows represent direction from the mean of the ancestral samples to the mean of the evolved samples in each divergence level.

We compared the changes in gene expression between ancestral and evolved populations in a principal component analysis (PCA). Consistent with an overall convergent expression change, we noticed that the direction of expression changes from ancestral to evolved replicates was shared across the divergence levels (Figure 2). This pattern was apparent across axes 2–4. PC axis 1 separated the species and explained 50% of the variation in the data (Figure S1). For a better visualisation of the parallelism, we used a vector analysis based on the mean of the ancestral and evolved populations for each level of divergence. We identified a common orientation of the vectors along which replicates evolved across all divergence levels (Figure 2A,B). If the angle between two vectors is 0°, they point in exactly the same direction and are considered parallel. Conversely, if the angle is 180°, the vectors point in opposite directions. The angle between the within‐ and between‐population vectors on PC axes 2 and 3 was 48° (Figure 2A). For the angle between the within population and between species vectors, it was 40° on PC axes 2 and 3 (Figure 2A). The last comparison, between the between populations and between species, had the smallest angle of 9°. The angles were much smaller when PC axes 3 and 4 were used (Figure 2B); 23° between the within‐ and between‐population vectors, 18° for the within population and between species vectors, and only 5° between the between populations and between species vectors (Figure 2B). Although this vector analysis is wide‐spread in the analysis of phenotypic parallelism, it is mostly descriptive as statistical testing is difficult (James et al. 2021).

### 2.4 Parallelism in Gene Expression Evolution Across Divergence Times

We compared the parallelism of gene expression evolution at three levels of divergence: within a population by comparing replicates from the same founder population, between geographically separated populations of the same species and between two species sampled at the same location on the same date. We used two different measures of parallelism to quantitatively assess parallelism in gene expression evolution: (i) the number of shared genes with significant differential expression and (ii) the correlation of the magnitude of change (log_2_FC).

**FIGURE 3:**
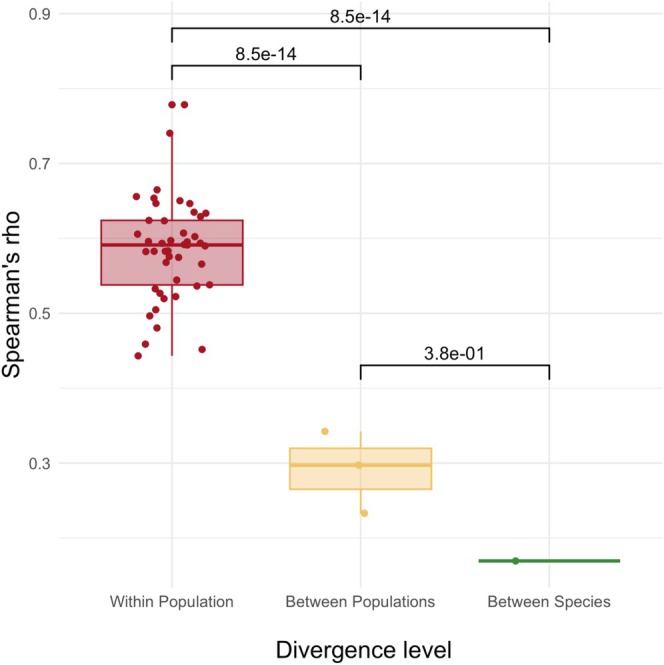
Spearman's *ρ* of the expression difference between the ancestral state and evolved one, measured in log_2_FC, from all possible pairwise comparisons within each of the three divergence levels: within population, between populations and between species. *p*‐values are from one‐sided Wilcoxon signed rank test (see Section 2).

Independent of the level of divergence, more genes shared a significant expression change during adaptation than expected by chance (Fisher's exact test *p* < 0.001, Table S2). The degree of parallelism varied with divergence between the groups compared, with the highest parallelism between replicates derived from the same founder population (within population) and the lowest parallelism between species. In all possible pairwise comparisons, between 410 and 1317 genes shared altered expression during the adaptation process at the level of within‐population divergence, yielding an average Jaccard Index of 0.28 (Figure 3). In all possible pairwise comparisons between populations, 151–180 genes were shared, resulting in Jaccard indices between 0.18 and 0.24 (Figure 3). The most divergent comparison between the two species, *D. simulans* and *D. melanogaster* , shared only 72 significantly differentially expressed genes, resulting in a Jaccard index of 0.10 (see Table S1 for the number of differentially expressed genes on the other divergence levels).

Comparing groups with different divergence levels based on the number of genes with a significant change in expression may give an incomplete picture because it relies on an ad hoc significance threshold rather than taking into account the magnitude of change. Therefore, we also measured parallelism using the correlation of log_2_FC (evolved vs. ancestral). Across all divergence levels, the expression changes were significantly correlated (Figure 2, Spearman's correlation test *p* < 0.001 for all pairwise comparisons). Similar to the analysis of shared significant genes, we found a higher correlation of log_2_FC within populations, followed by the comparison between populations. The lowest correlation was observed for the comparison between species (Figure 3).

### 2.5 Parallel Evolution of Biological Functions Across Divergence Time

**FIGURE 4:**
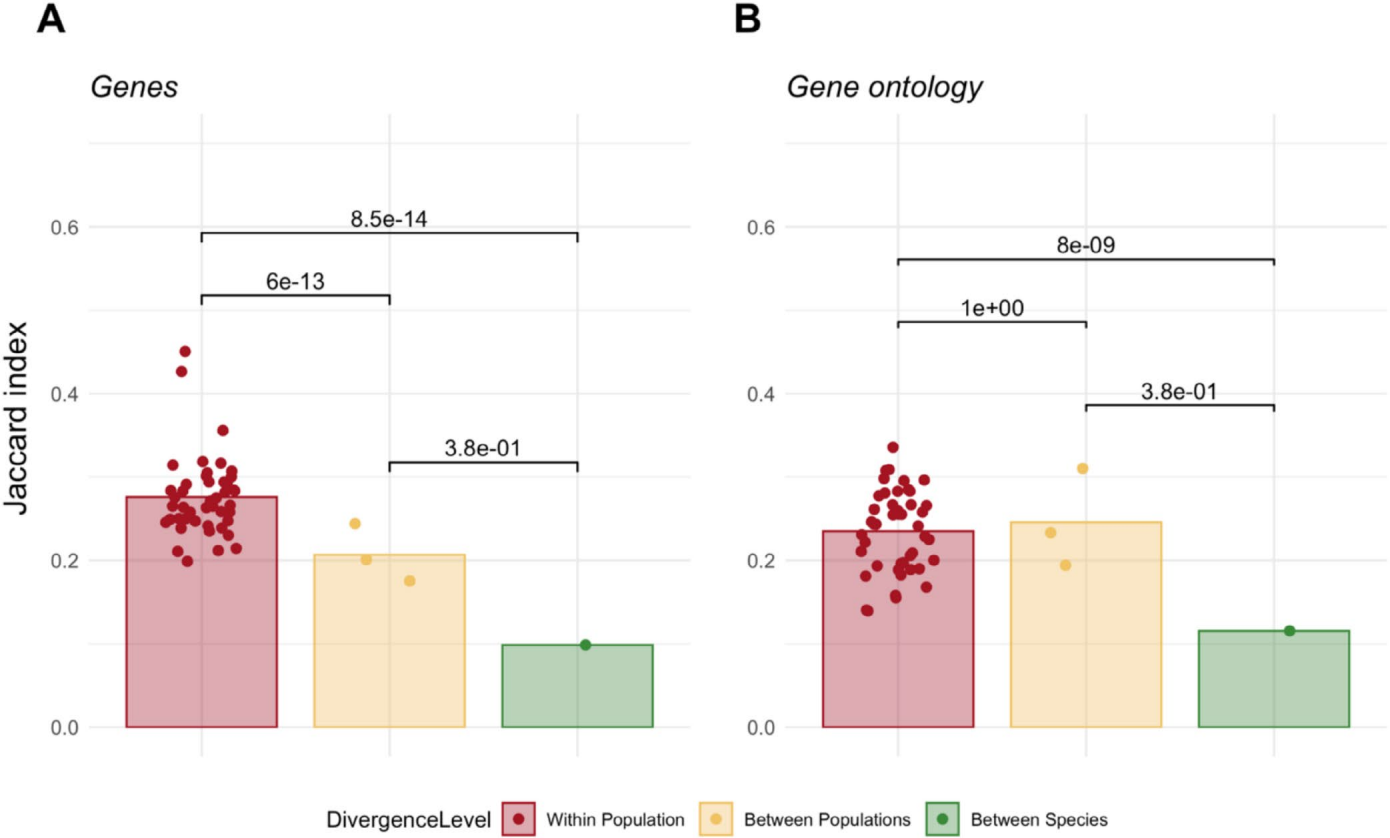
Jaccard indices from all pairwise comparisons within each of the three divergence levels; within population; between populations and between species, of (A) differently expressed genes between the ancestral state and evolved one and (B) enriched gene ontology terms. *p*‐values are from one sided Wilcoxon signed rank test (see Section 2).

**FIGURE 5:**
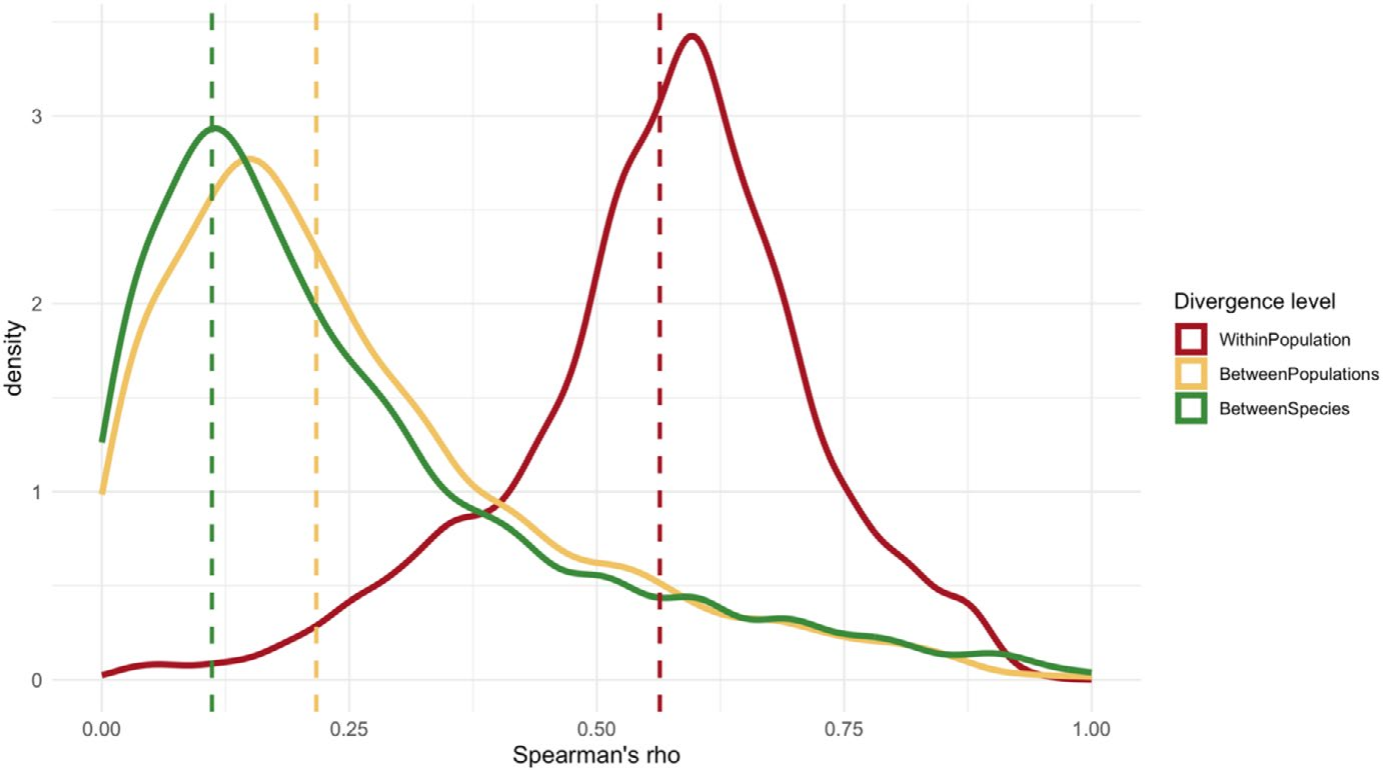
Characterisation of parallelism across gene ontology terms based on pairwise correlations. Distributions of Spearman's *ρ* across all genes in a given GO term in each divergence level. The dashed lines represent the mean of each distribution. In the case of between populations and within population, more than one pairwise comparison was performed for each GO term due to the availability of multiple replicates. Therefore, we averaged all correlation coefficients per GO term. Sampling one random correlation coefficient from a single pairwise comparison for each GO term resulted in the same pattern (Figure S6).

We used the GO to estimate the parallelism of the evolved biological processes. We calculated the Jaccard Index of significantly enriched GO terms in pairwise comparisons within each divergence level (Figure 4B). Consistent with previous studies (Lai et al. 2023), there is a trend toward higher levels of parallelism at higher levels in the biological organisation hierarchy (Figure 4). The analysis of parallel evolution of biological processes was based on shared enriched GO terms, which depend on ad hoc significance cutoffs for both gene expression differences and GO enrichment. Therefore, we included another analysis that does not depend on significance cutoffs. For each divergence level, we determined the correlation (Spearman's *ρ*) of the gene expression difference (log_2_FC) for all genes belonging to a given GO term. The distribution of the mean correlation coefficient from all possible pairwise comparisons in the within‐ and between‐population divergence level, and the single comparison for the within species level, based on 3968 GO terms is shown in Figure 5. The overall pattern was similar to the previous analyses, with a decreasing parallelism with increasing distance. However, the shape of the distribution was quite different for the within‐population comparison. Although the between species and between populations comparisons had a long tail with some GO terms having a high correlation, the distribution of the within‐population comparison was symmetric. Between species and between populations comparisons had a very similar, but low parallelism for most GO categories, whereas for within‐population comparisons most GO categories exhibited a rather high parallelism. The persistent discrepancy in the parallelism of evolution at the level of biological processes between differently diverged lineages indicates that the lineages have adapted to temperature through a combination of shared and distinct routes.

## 3 Discussion

Parallel evolution has been observed across a diverse range of taxa, where the same genes or genomic regions frequently contribute to adaptation to similar environmental stressors in different lineages, even with varying degrees of divergence (Colosimo et al. 2005; Elmer et al. 2014; Martin and Orgogozo 2013; Rougeux et al. 2019; Soudi et al. 2023; Stern 2013; Yeaman et al. 2016). This parallel evolution is important because it provides robust evidence for the adaptive importance of evolutionary changes.

Of particular interest are, however, those cases in which populations or species adapt to the same environment by different means (Barghi et al. 2019; Bolnick et al. 2018; Poore et al. 2023). This insight was explained by genetic redundancy, whereby changes at distinct genetic pathways nevertheless facilitate the evolution of the same adaptive trait (Barghi et al. 2019, 2020; Láruson et al. 2020). Thus, it is important to note that even non‐parallel evolution does not preclude the possibility that different groups adapt through similar phenotypic changes.

This insight raises the question of why parallel evolution occurs in some cases and not in others. Theory on polygenic adaptation has been instrumental in identifying several key factors, including the complexity of the trait (i.e., the number of contributing loci), the distance to trait optima, the effect size of contributing loci, genetic drift and allele frequencies (Barton 1989; Barton et al. 2017; Devi and Jain 2023; Franssen et al. 2017; Hayward and Sella 2022; Höllinger et al. 2019; Jain and Stephan 2017). Nevertheless, empirical studies investigating the influence of different factors on parallel evolution remain scarce.

Evolve and resequencing (E&R) is an excellent approach to study parallel evolution. In contrast to natural environments, experimental evolution in the laboratory allows for the precise control of environmental variables, thus eliminating the confounding effects of different co‐varying environmental factors during adaptation (Kawecki et al. 2012). Consistent with genetic redundancy, non‐parallel genomic selection responses have been described in several experiments starting from a polymorphic founder population (Barghi et al. 2019; Bolnick et al. 2018; Griffin et al. 2017; Schlötterer 2023), although the degree of parallelism varies between experiments (Barghi et al. 2019; Burke et al. 2010; Graves et al. 2017). Studies of parallel evolution using genetically divergent founder populations have yielded highly controversial results—either very high parallel response at the phenotypic and genetic level (Graves et al. 2017) or parallelism at the phenotypic but not genetic level (Johnson et al. 2022, 2023). The reason for this discrepancy between these studies is not yet fully understood, but it has been proposed that variation in linkage disequilibrium may be a major factor determining the degree of parallelism at the genomic level (Griffin et al. 2017; Sachdeva and Barton 2018; Schlötterer 2023).

Inconsistent (non)‐parallel responses in gene expression levels have been shown for populations evolving either under shared selection stressors or in combination with parallel changes of high‐level phenotypes (Durkin et al. 2024; Jacobs et al. 2020; Szukala et al. 2023; Wos et al. 2021). It is, however, not clear if these non‐parallel gene expression responses are the outcome of redundancy or can be attributed to environmental heterogeneities, which are typically unavoidable in naturally evolving populations. This study used experimental evolution to adapt groups of replicate populations with different levels of divergence to the same environmental conditions. The important difference from previous analyses of the influence of genetic divergence on parallel responses is that this study did not compare the similarity of adaptation that has occurred in the wild, which is difficult to standardise. Rather, we started with genetically diverged groups and evolved them under identical culture conditions to minimise the confounding effects of genotypic differences and uncontrolled environmental heterogeneity. Anticipating that the combination of *cis*‐ and *trans*‐effects may make gene expression less sensitive to genetic divergence, we tested the parallel response in gene expression across three levels of genetic divergence: replicates from the same founder population, different founder populations and two closely related species.

Our results show that the divergence between groups has a strong effect on the degree of parallelism in gene expression evolution. Previous work has already documented non‐parallel gene expression evolution among replicates from the same founder populations (Lai et al. 2023; Lai and Schlötterer 2022), suggesting redundancy for gene expression. Thus, adaptation to the novel environment may be achieved by different combinations of gene expression changes. The decreasing degree of parallelism for gene expression evolution with increasing divergence suggests that although the polygenic basis of gene expression could lead to genetic redundancy, it does not mean that across different levels of divergence all expression changes are equally likely. Rather, we propose that with increasing divergence of populations also the effect size and allele frequency of the genetic variants determining gene expression levels are becoming increasingly diverged. Hence, although adaptation can be achieved through different combinations of gene expression changes (redundancy), which adaptive paths of gene expression evolution are being used depends on the characteristics of each population that diverge with genetic distance.

It is likely that the founder populations with different geographic origins exhibited not only allele frequency differences by drift but also adaptations to disparate habitats. This suggests that the distance to the new trait optimum imposed by the laboratory conditions may differ among them. As a result, the degree of parallelism between populations of different geographic origins may be reduced. In order to minimise the influence of different distances to the new trait optimum, we conducted an interspecific comparison using two founder populations that were collected on the same date and location. Nevertheless, it cannot be excluded that adaptation to the same environment does not result in different distances to the new trait optimum in the laboratory. Moreover, the level of polymorphism and linkage disequilibrium may be additional confounding factors.

We also note that not all populations evolved for the same number of generations. If the quantification of redundancy depends on the duration of the experiment, with some populations having reached trait optimum but others not, this could affect our conclusions by suggesting a lower parallelism. Nevertheless, for three reasons we do not think that this is a relevant concern for the interpretation of our data. (1) The between‐species comparison involved populations that evolved for the longest time in the laboratory but showed the least parallel selection response. (2) Under the selective sweeps paradigm, genetic drift could delay/enhance the spread of a favourable allele. This makes the inference of parallel selection responses time‐dependent, as heterogeneity created by genetic drift will be levelled out at later generations—as long as the favoured alleles are retained in the population. With genetic redundancy, genetic drift during the first generations also delays/enhances the frequency increase of contributing alleles after a shift in trait optimum. The key difference is, however, that because not all alleles are needed to reach the new optimum, stochastic events at the first generations determine which alleles will be used to contribute to trait optimum. Unlike selective sweeps, alleles disfavoured during the first generations are unlikely to contribute to adaptation because this will be achieved by those alleles that increased in frequency through the combined effects of selection and drift. This implies that the inference of parallelism with redundancy is not very sensitive to differences in the number of generations of evolution in the new environment. (3) Although the dynamics of gene expression changes during experimental evolution are not yet well studied, it is known that most allele frequency changes occur at the beginning of the experiment (Christodoulaki et al. 2022). These slow allele frequency changes at advanced generations are fully consistent with the expectations for populations that have reached trait optimum (Barghi et al. 2020; Franssen et al. 2017; Hayward and Sella 2022). Hence, it is likely that all populations studied have evolved for a sufficient number of generations to be close to the trait optimum for the new laboratory environment. Although we do not consider it likely that it applies to our experiments, we would like to highlight another property of polygenic adaptation, which also affects parallelism. Three different phases of allele frequency changes can be observed during polygenic adaptation (Barghi et al. 2020; Franssen et al. 2017). The first phase is characterised by a rapid allele frequency change. As populations are approaching the new trait optimum, allele frequency changes slow down, indicating the transition to the second phase, which is characterised by plateauing allele frequencies. The third phase exhibits increased allele frequency changes, which are driven by stabilising selection reducing fitness variation in the population. This implies that parallelism will be reduced during the third phase (Barghi and Schlötterer 2020). Although in small populations the third phase is reached faster, we consider it unlikely that this phase has been reached in our experiments because no acceleration in allele frequency changes has been observed in the genomic analyses of later generations (Christodoulaki et al. 2022).

One limitation of our study was the different number of observations at the different divergence levels. Nevertheless, the difference in parallelism between experiments with the same founder population and different founder populations persisted, even when we reduced the data to the same number of observations. The analysis on the species level was restricted to a single species pair, which exhibited less parallelism than any pairwise comparison on the lower divergence levels. Although we acknowledge that the inclusion of more species pairs could have provided additional confidence, we also caution that even if additional species pairs with similar divergence levels can be sampled, habitat differences may contribute to even less parallelism due to local adaptation. Therefore, we opted for a single species pair sampled at the same location.

A further interesting question would be to determine the point at which trait hierarchy or network position will be no longer affected by genetic divergence such that all groups will show full parallel selection responses. A parallel response, not affected by genetic divergence, suggests that no alternative paths can be used, and therefore it is very likely that this point coincides with the actual target of selection. Some data from replicate *D. simulans* populations derived from the same founder population indicate that metabolites exhibit a higher degree of parallelism than gene expression (Lai et al. 2023). Nevertheless, the non‐parallel evolution of metabolites among replicates remains still considerable (Lai et al. 2023). We can, however, not rule out that populations/species use entirely different traits to adapt to a new environment, which implies that convergence would occur only at the level of fitness, not at a high level trait. Nevertheless, the consistent response of replicate populations for high level phenotypes related to metabolism and fecundity (Barghi et al. 2019; Mallard et al. 2018) strongly suggests that adaptive convergence occurs at a higher trait level.

The non‐parallel selection response is in strong alignment with the omnigenic model (Boyle et al. 2017). The hypothesis that only a few key genes are directly responsible for complex traits is expanded to suggest that almost all genes expressed in relevant cell types can influence these traits to some degree. It is possible that individual genes and their expression are not the direct target of selection. In contrast, selection acts on a higher‐order phenotype, composite traits that result from the intricate interactions of numerous genes. These higher‐order phenotypes can be modified through multiple pathways within the regulatory networks. Consequently, the majority of genes can exert an influence on complex traits, either directly or indirectly, through their participation in these extensive and interconnected regulatory networks. This broad influence implies that genetic variation in a wide range of genes, even those with seemingly minor effects, can collectively shape phenotypic outcomes. This perspective is consistent with our findings, which indicate that selection may target the overall regulatory architecture rather than individual genes. This allows for a diverse array of genetic routes to similar adaptive phenotypes.

## 4 Materials and Methods

### 4.1 Experimental Evolution

We explored the parallelism of gene expression evolution using experimental evolution in the same high‐temperature laboratory environment across three levels of divergence: (1) between replicate populations from the same *D. simulans* founder population, representing within‐population comparison; (2) between *D. simulans* populations from different continents (Europe: Portugal; North America: Florida and Africa: South Africa) and (3) between two species, *D. melanogaster* and *D. simulans* , collected at the same site in Portugal (Figure 1). For each of the experiments, isofemale lines from each population were kept in the laboratory for about five generations before the experiment started. Each replicated founder population was created from the same number of mated females from each isofemale line. Since the number of isofemale lines differed between experiments, but the founder populations were of equal size, this implies that the number of females used from each isofemale line differed between experiments. Each replicate was exposed to the same laboratory selection regime with fluctuating high temperature, alternating between 12 h in the dark at 18°C and 12 h in light at 28°C, mimicking the diurnal cycle. The census population size of the evolving populations was 1000–1250, whereas the isofemale lines were kept at ~50 flies per vial at 18°C as stock for future reconstitution of ancestral populations (Figure 1). The evolved populations from *D. simulans* Florida (Barghi et al. 2019), *D. simulans* Portugal (Mallard et al. 2018) and *D. melanogaster* (Tobler et al. 2015) have already been studied before.

### 4.2 Common Garden Experiments

Given the scale of the experiment, it was logistically not possible to measure gene expression in a single common garden. Therefore, we performed separate common gardens for each level of divergence: (1) within population: 10 evolution replicates derived from a single *D. simulans* population collected in Florida; (2) between populations: three different *D. simulans* founder populations collected in Florida, Portugal and South Africa; (3) between species: *D. simulans* and *D. melanogaster* founder populations originating from the same geographic location (Portugal) (Figure 1). Two populations ( *D. simulans* Florida and Portugal) are measured in two independent common garden experiments. They cannot be compared due to random uncontrolled confounding factors of the experiments. Despite our aim to use a constant environment across common garden experiments, minor uncontrolled factors, such as humidity, may introduce variability between experiments. Additionally, different RNA‐Seq library preparation protocols and sequencing platforms can introduce technical noise, further complicating direct comparisons. However, this noise is random across experiments and therefore cannot account for our key observation—the decline in parallel gene expression responses with increasing divergence. Environmental factors within each common garden experiment are negligible, as all flies were maintained under identical conditions prior to phenotyping. Therefore, a direct comparison within each common garden experiment provides the most reliable approach. The within‐population experiment has been described previously (Hsu, Jakšić, et al. 2020; Lai and Schlötterer 2022). The same protocol was used for the other two experiments, the between populations and between species comparisons. In brief, the ancestral populations for each evolution experiment were reconstituted by pooling an equal number of females from each of the available isofemale lines. We note that no significant genetic differences are observed between a reconstituted ancestral population and the original ancestral population (Nouhaud et al. 2016). Limited impact is expected from new mutations occurring during the maintenance of the isofemale lines, because they occur only in a single isofemale line. The large number of isofemale lines used results in a low frequency of new mutations in the reconstituted population. Furthermore, almost all new mutations will be heterozygous in the reconstituted ancestral population. Given that novel mutations are typically recessive (Charlesworth and Charlesworth 2010), their influence on gene expression can be neglected (Lai et al. 2024). The between populations and between species experiments included five independently evolved replicates for each population and the corresponding reconstructed ancestral populations (Figure 1), which were treated in a common garden environment matching the conditions during experimental evolution. Ten independently evolving replicates were used for the within‐population comparison, with three sub‐replicates sampled for each independently evolving replicate and a total of five ancestral replicates. All three common garden experiments were controlled for density, with 400 eggs per bottle in the same temperature regime as during the evolution experiment for two generations before sampling, to avoid transgenerational effects on the transcriptional profiles. At the second generation, freshly eclosed males were allowed to mate randomly until they were separated from females under CO_2_ anaesthesia and recovered separately for 2 days, in batches of 50 males per vial. At the age of 4–5 days, 50 males per replicate were snap frozen and stored at −80°C until RNA extraction. Three sub‐replicates were collected for the within‐population experiment that enables the comparison between evolution replicates. A previous study found that females exhibited a greater allometric difference than males after adaptation (Hsu, Jakšić, et al. 2020). Therefore, the focus of this study is on males.

### 4.3 RNA Extraction and Sequencing

For all common garden experiments, samples of 50 males for each replicate were taken from −80°C storage and immersed and homogenised immediately with Qiazol (Qiagen, Hilden, Germany). RNA was extracted from whole body flies using the Qiagen RNeasy Universal Plus Mini kit. RNA‐seq libraries were prepared with the TrueSeq standard mRNA library kit on a Neoprep device (software version 1.1.0.8 and protocol version 1.1.7.6, Illumina, San Diego, USA). The starting concentration of RNA was 100 ng and default settings for an insert size of 200 bp and 15 PCR cycles were used. Libraries were randomised across Neoprep library cards with identical lot numbers for each data set (A) *D. melanogaster* Portugal from the between species common garden experiment, (B) *D. simulans* Portugal from *the between* species common garden experiment, (C) *D. simulans* Portugal, Florida and South Africa from the between populations common garden experiment and (D) *D. simulans* Florida samples from the within‐population common garden experiment. 50 bp reads were sequenced on the Illumina HiSeq 2500 platform. Because RNA‐Seq libraries for the between species dataset were generated with different lot numbers, we analysed the transcriptome separately for each species and then determined the parallel gene expression evolution.

### 4.4 Sequence Data Processing

We used a standard pipeline for all datasets as described in Hsu, Belmouaden, et al. (2020). Briefly, the sequenced reads were trimmed using ReadTools (version 1.5.2) (Gómez‐Sánchez and Schlötterer 2018), with a quality threshold of 20. The *D. simulans* reads were mapped with GSNAP (version 2018‐03‐25) (Wu and Nacu 2010) to a *D. simulans* reference genome (Palmieri et al. 2015), where the annotation was based on a *D. melanogaster* annotation version 5.49. Therefore, the *D. melanogaster* reads were mapped with the same mapper to the same version from FlyBase (5.49), avoiding issues caused by inconsistent annotations when comparing the evolutionary response between species. The following parameters were used for mapping all datasets: ‐A: SAM, ‐k: 15, ‐N: 1, ‐m: 0.08. 3′‐bias was evaluated using RSeQC (Wang et al. 2012). Rsubread (version 2.2.2) (Liao et al. 2019) was used for quantifying reads.

We constrained our analysis to orthologous genes between *D. simulans* and *D. melanogaster* with a minimum expression of one count per million reads in all samples, resulting in a total of 9914 genes. We obtained on average 11.7 million counts per sample, with a range of 6.4–15.7 million counts per sample. Downstream analyses were performed in R (version 4.0.2). Differential expression analysis was carried out with edgeR (Robinson et al. 2009).

### 4.5 Statistical Analysis

To evaluate the evolutionary response in expression for each gene, a linear model was fitted using the function glmFit() in edgeR, where normalised gene expression for each gene is the response variable and the evolutionary state is the explanatory variable: expression = evolution + ℇ. Contrasts between evolved and ancestral samples (Figure 1) were performed using the function glmLRT(). Correction for multiple testing was done according to Benjamini and Hochberg's FDR correction (Benjamini and Hochberg 1995). The directionality of the evolutionary expression change for significant genes was determined by the log_2_ scaled fold change (log_2_FC).

We assessed the evolutionary response on a higher biological organisation level than the expression of individual genes, by a GO enrichment analysis of significantly differentially expressed (DE) genes. The GO enrichment analyses were done using the package topGO (Alexa and Rahnenfuhrer 2020), precisely with the ‘Weighted01’ algorithm, which is aware of the GO hierarchy.

PCA analysis was performed on normalised logarithmic transformed CPM values. To estimate the direction of phenotypic evolution in the PC dimensions, we followed James et al. (2021) by using the angle between vectors to estimate the degree of parallelism. The vectors were constructed from the mean of the ancestral samples to the mean of the evolved samples for each divergence level independently. By elementary trigonometry, the (acute) angles between two lines with slope *m*_1_ and *m*_2_ are given by

(1) $$\text{angle}=\arctan\left(\left|\frac{m_{2}-m_{1}}{1+m_{1}*m_{2}}\right|\right)$$

We calculated the angles between vectors of each divergence level in a pairwise comparison using Equation (1), where *m*_1_ is the slope of one divergence level and *m*_2_ to the slope of the second. The angle radians were converted to degrees by multiplying them with $\frac{180}{\pi }$.

To quantify the parallel evolution response, we calculated the Jaccard index (Equation 2) of the overlap between significant DE genes in each pairwise comparison for each divergence level. The same procedure was applied to GO terms that were enriched. To determine if more genes/GO terms were shared between the two groups compared than expected by chance, we applied Fisher's exact test. Additionally, to avoid a fixed significance cutoff and to account for the magnitude of changes, we calculated Spearman's *ρ*. This allowed us to assess the correlation between the log_2_FC across the entire transcriptome, capturing the similarity in the magnitude of expression evolution.

(2) $$J\left(A,B\right)=\frac{\left|A\cap B\right|}{\left|A\cup B\right|}$$

To statistically compare the degree of parallelism between different levels of divergence, estimated with *ρ* and Jaccard Index (Equation 2), we used the Wilcoxon signed‐rank test. The degree of parallelism was compared on three levels: (1) within‐population and the average of the between‐populations estimates, (2) within‐population and the single estimate of the between species and (3) between populations and the single between species estimate. Bonferroni correction was applied to account for multiple testing (Bonferroni 1936).

The number of samples differed across the datasets (the three divergence levels), which resulted in a varying number of possible pairwise comparisons (within population: 45; between populations: 3; between species: 1; Figures 1, 2, 3). To ensure that the results were not affected by this, we performed jackknifing of both the Spearman's *ρ* and the Jaccard Index comparison between divergence levels for genes and GO terms. We formed all possible combinations of 3 out of the 10 replicates of the within‐population dataset and determined the test statistics (correlation/Jaccard Index) for each Jackknifing. To obtain a *p*‐value, we counted the number of times that the jackknifed estimate exceeded the values of either the average of the between populations estimates or the between species estimate and divided the counts by the number of jackknifed iterations. The results from this procedure all align with the conclusions of the Wilcoxon test (see Figures S3 and S4). It is important to note that the data structure varied between the three different divergence levels. The within‐population data included sub‐replicates and a higher number of evolutionary replicates, whereas the other two divergence levels lacked sub‐replication and had fewer evolutionary replicates (see Table 1 and Figure 1). Differential expression analysis using five evolutionary replicates reduces the effect of drift, as the changes need to be relatively consistent between the evolutionary replicates to reach significance. This is one way of controlling for drift in gene expression analysis. On the other hand, sub‐replicates do not control for genetic drift, as they are generated from the same evolved replicate only for phenotyping. If expression patterns are correlated, drift will add noise and therefore reduce similarities in the within‐population comparisons. As the within‐population divergence level has the highest degree of parallelism, we consider the results to be conservative (Figures 3 and 4). We also note that the populations evolved for a different number of generations to the same new environment (Table 1). The South African *D. simulans* populations stand out with the fewest generations of evolution. Despite this, the South African populations behave similarly to the populations from Florida and Portugal when considering the correlation coefficient, the Jaccard Index, or the degree of evolutionary gene expression change from the ancestral to the evolved populations (Figure S2B). Therefore, we do not expect that the difference in the number of generations in the new environment affects the interpretation of our results.

To assess parallelism across GO terms without the restriction of a significance cutoff, we utilised Spearman's correlation coefficient (*ρ*) of the evolved gene expression change (log_2_FC ancestor vs. evolved) in sets of genes that were included in a given GO term. We did this for all GO terms, a total of 3968 terms. We performed pairwise comparisons within each divergence level, which resulted in a different number of data points per GO term for each divergence level. For the within‐ and between‐population data, this resulted in 45 and 3 data points per GO term, respectively. Since the between species data consisted of a single data point per GO term, we averaged pairwise correlations per GO term for the other two divergence levels. Similar results were obtained by randomly sampling one pairwise comparison per GO term (Figure S6). We examined the distribution and mean across all GO terms within each divergence level to identify GO terms with high parallelism in each divergence level.

Visualisation was done on these measures on gene expression and GO, with the R package ggplot2 (Hadley 2016).

## Author Contributions

D.A.V.T., S.K.H. and C.S. conceptualised the study and wrote the manuscript. D.A.V.T. and S.K.H. carried out the analysis. V.N. prepared the RNA‐seq libraries used in this study and oversaw the common garden experiments and supervised the maintenance of the experimental evolution populations.

## Disclosure

Benefits Generated: Benefits from this research accrue from the sharing of our data and results on public databases as described above.

## Conflicts of Interest

The authors declare no conflicts of interest.

## Supporting information


Data S1.

Appendix S1.

## Data Availability

The within‐population data have been published previously (Hsu, Jakšić, et al. 2020; Lai and Schlötterer 2022), as well as the between species (Hsu, Belmouaden, et al. 2020; Thorhölludottir et al. 2023). The sequenced reads for the between population experiment will be made available upon publication in the European Sequence Archive (https://www.ebi.ac.uk/ena), under the accession number PRJEB79716. Filtered read count tables from all experiments and scripts for analysis will be available on the GitHub repository for this study upon publication: https://github.com/DagnyAsta/ReducedParallelGeneExpressionEvolutionWithIncreasingGeneticDivergence.
