# Supplementary material for: Reduced Parallel Gene Expression Evolution With Increasing Genetic Divergence—A Hallmark of Polygenic Adaptation
Source: Mol Ecol. 2025 May 16;34(12):e17803. doi: 10.1111/mec.17803 (PMC12143366; doi:10.1111/mec.17803)
Supplement: Supplementary file 2 — Appendix S1. [file MEC-34-e17803-s002.pdf]

Supplementary

Supplementary Table 1 . Number of up- and down regulated gene in each population under study

**Contrast**	**Upregulated genes (FDR < 0.05)**	**Downregulated genes (FDR < 0.05)**
FL ancestor – FL evolved 1	622	710
FL ancestor – FL evolved 2	599	714
FL ancestor – FL evolved 3	363	531
FL ancestor – FL evolved 4	474	589
FL ancestor – FL evolved 5	448	559
FL ancestor – FL evolved 6	572	782
FL ancestor – FL evolved 7	1106	1145
FL ancestor – FL evolved 8	1089	1966
FL ancestor – FL evolved 9	992	1065
FL ancestor – FL evolved 10	645	693
FL ancestor – FL evolved	152	252
PT ancestor – PT evolved	387	323
SA ancestor – SA evolved	183	183
D. sim PT ancestor – D. sim PT evolved	207	190
D. mel PT ancestor – D. mel PT evolved	244	162

Supplementary Table 2. Summary statistics and p-values from Fisher's exact test from all pairwise comparisons within each divergence level, on sets of differently expressed genes in each replicate/population/species.

DivergenceLevel	OR	pval	padj
**WithinPopulation**	10.72	3.35e-253	2.74e-252
**WithinPopulation**	9.73	1.45e-184	2.54e-184
**WithinPopulation**	7.65	8.54e-164	1.07e-163
**WithinPopulation**	8.08	3.23e-167	4.28e-167
**WithinPopulation**	9.31	1.11e-230	4.93e-230
**WithinPopulation**	5.14	1.15e-151	1.37e-151
**WithinPopulation**	7.09	1.71e-214	5.58e-214
**WithinPopulation**	7	5.59e-209	1.52e-208
**WithinPopulation**	6.86	4.36e-168	6.10e-168
**WithinPopulation**	12.9	1.28e-231	6.26e-231
**WithinPopulation**	10.13	3.13e-211	9.01e-211
**WithinPopulation**	11.43	5.83e-227	2.04e-226
**WithinPopulation**	10.08	6.10e-242	3.74e-241
**WithinPopulation**	6.5	9.65e-192	1.97e-191
**WithinPopulation**	8.12	1.70e-236	9.23e-236
**WithinPopulation**	6.83	4.77e-197	1.02e-196
**WithinPopulation**	7.41	7.81e-178	1.20e-177
**WithinPopulation**	10.85	1.83e-183	3.09e-183
**WithinPopulation**	14.44	6.33e-228	2.39e-227
**WithinPopulation**	9.69	5.13e-185	9.30e-185
**WithinPopulation**	6.77	8.41e-151	9.82e-151
**WithinPopulation**	9.14	4.01e-198	8.94e-198
**WithinPopulation**	8.06	3.71e-176	5.50e-176
**WithinPopulation**	8.86	4.11e-169	5.92e-169
**WithinPopulation**	11.38	2.66e-206	6.86e-206
**WithinPopulation**	8.66	2.09e-187	3.93e-187
**WithinPopulation**	5.28	6.52e-132	7.44e-132
**WithinPopulation**	7.46	4.18e-190	8.20e-190
**WithinPopulation**	6.61	3.76e-166	4.85e-166

**WithinPopulation**	9.31	8.19e-200	1.91e-199
**WithinPopulation**	9.8	2.28e-203	5.59e-203
**WithinPopulation**	4.86	4.08e-114	4.45e-114
**WithinPopulation**	7.5	5.95e-183	9.71e-183
**WithinPopulation**	6.93	1.86e-167	2.54e-167
**WithinPopulation**	11.25	7.83e-229	3.20e-228
**WithinPopulation**	5.12	9.36e-153	1.15e-152
**WithinPopulation**	8.31	9.79e-253	6.85e-252
**WithinPopulation**	9.09	1.02e-270	1.25e-269
**WithinPopulation**	10.56	4.05e-259	3.96e-258
**WithinPopulation**	11.48	0.00e+00	0.00e+00
**WithinPopulation**	7.32	1.39e-295	2.27e-294
**WithinPopulation**	3.77	1.62e-99	1.69e-99
**WithinPopulation**	14.45	0.00e+00	0.00e+00
**WithinPopulation**	5.98	1.86e-179	2.93e-179
**WithinPopulation**	7.09	2.30e-212	7.05e-212
**BetweenPopulation**	11.49	7.36e-88	7.52e-88
**BetweenPopulation**	25.77	2.99e-120	3.33e-120
**BetweenPopulation**	16.45	1.09e-112	1.16e-112
**BetweenSpecies**	6.09	5.63e-28	5.63e-28

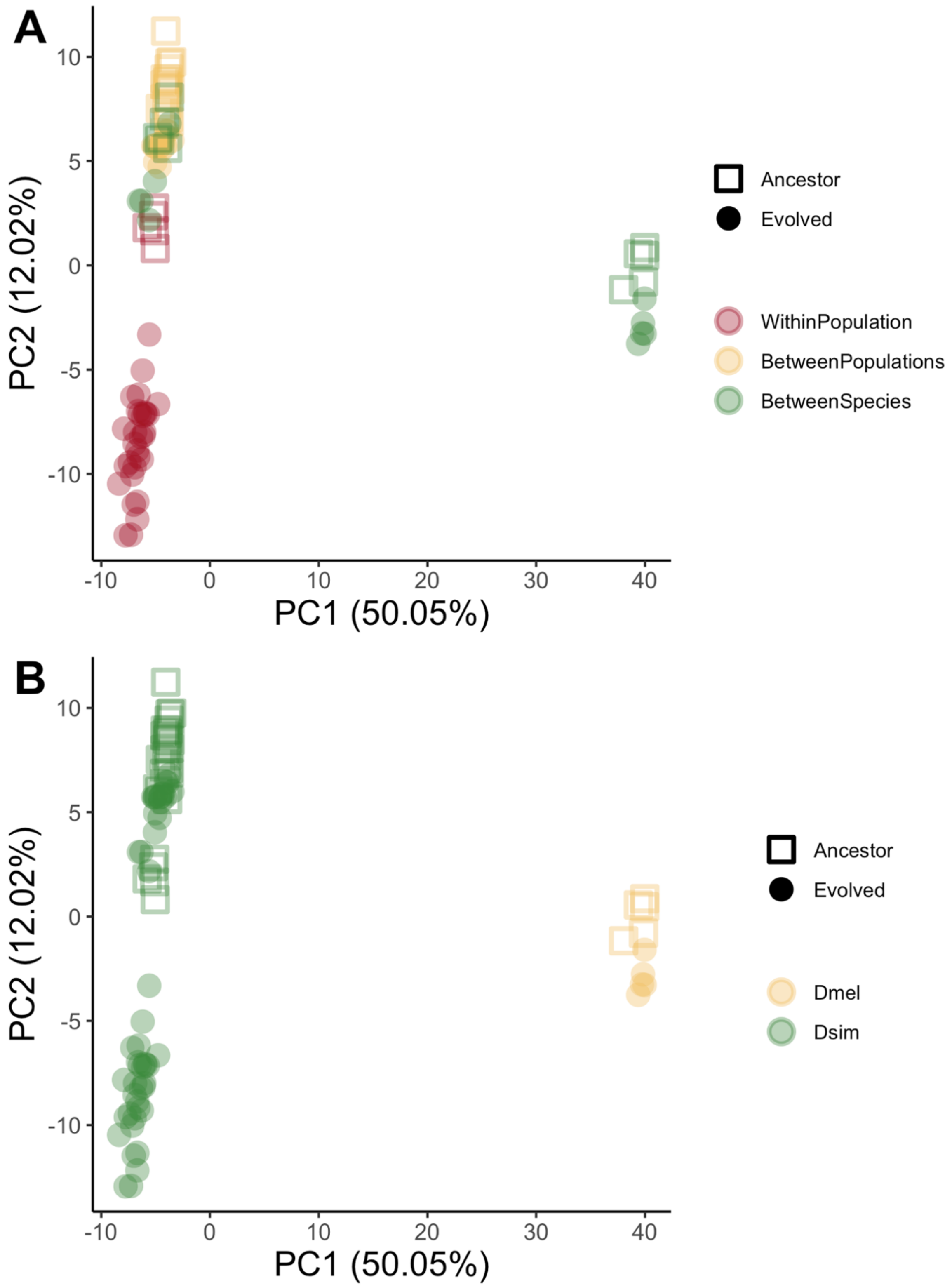

Supplementary Fig 1. We used Principal Component Analysis to determine the parallel response of the transcriptome to the same stressor on three different levels of divergence. The axis show PC1 and PC2 which are separating ancestors (open squares) from evolved (filled circles), the colours indicate the three divergence levels in pannel A, and the two species in B. PC1 separated the species.

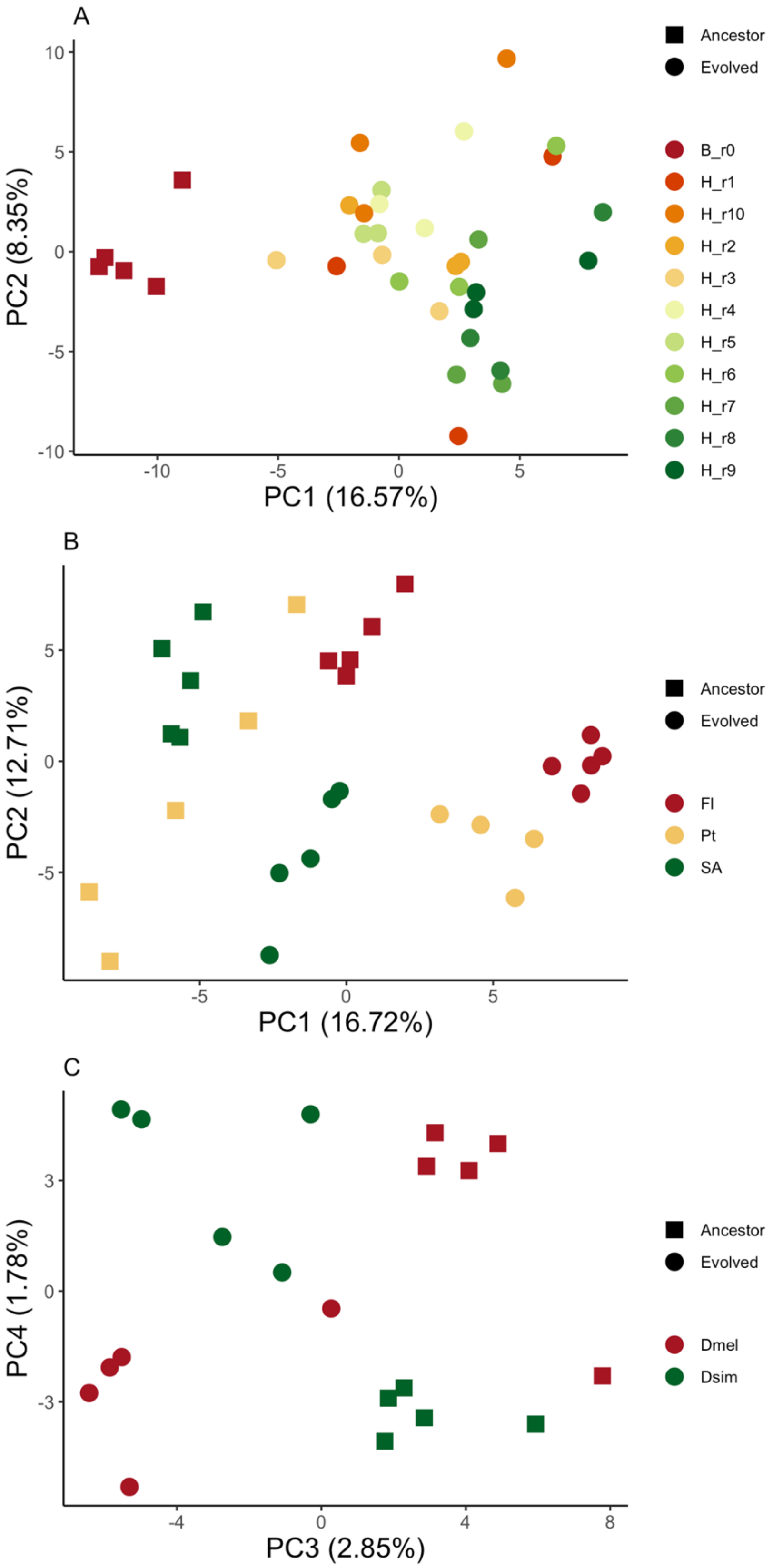

Supplementary Fig 2. Principal component analysis for all three divergence levels. A) between replicates, B) between populations and C) between species (note that here PC3 and 4 show the evolutionary response).

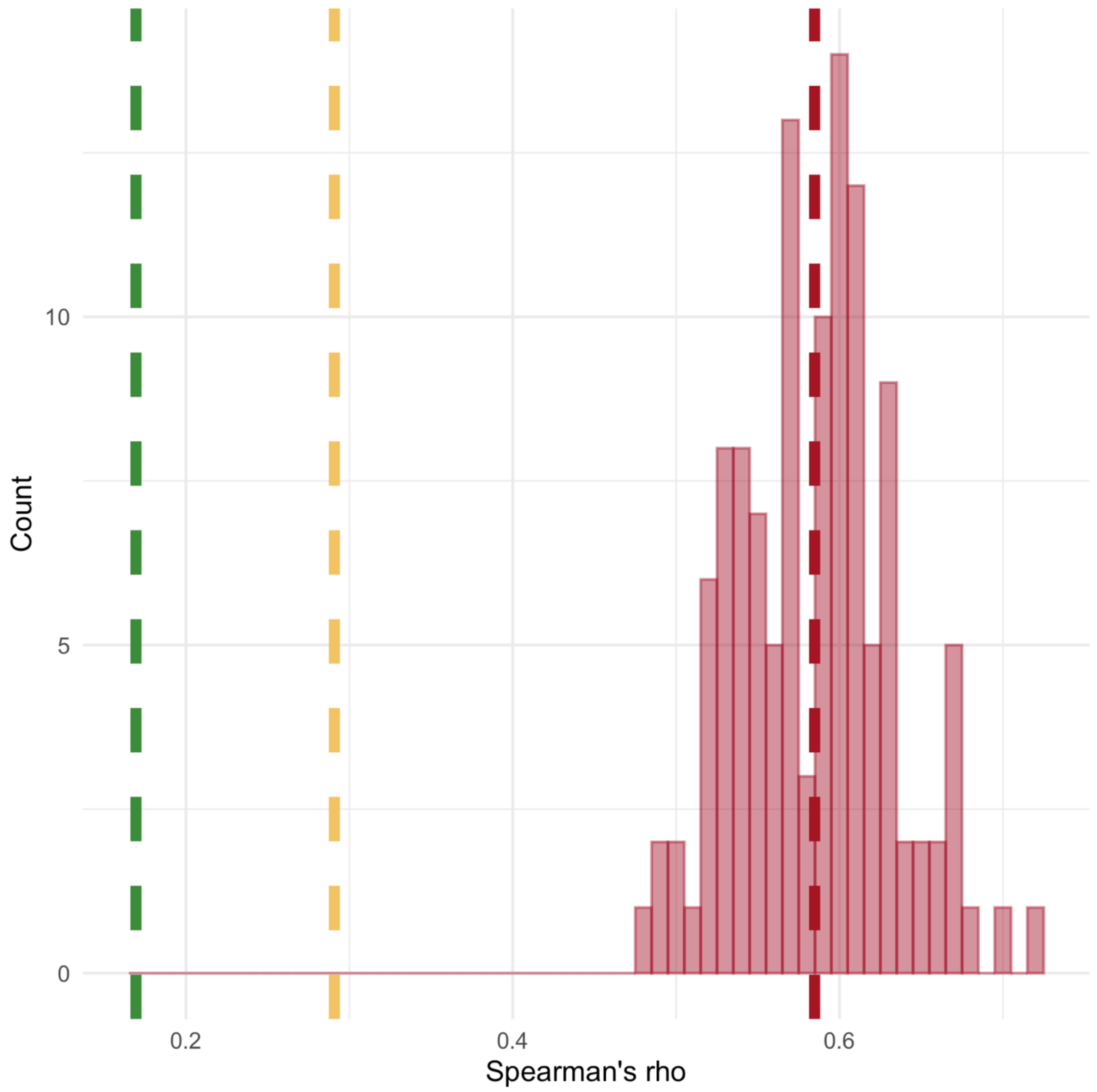

Supplementary Fig 3. Permuted Spearman's *rho* from all possible comparisons of 3 subsampled replicates from the within population data, where all possible combination of 3 replicates out of the 10 make up the permutations. The dashed purple line represents the mean of the distribution, while the blue is the mean value for the between population data and the green is the value for the between species data. Both means of between species and populations are significantly different from the permuted distribution of the within population data ($p = 0$).

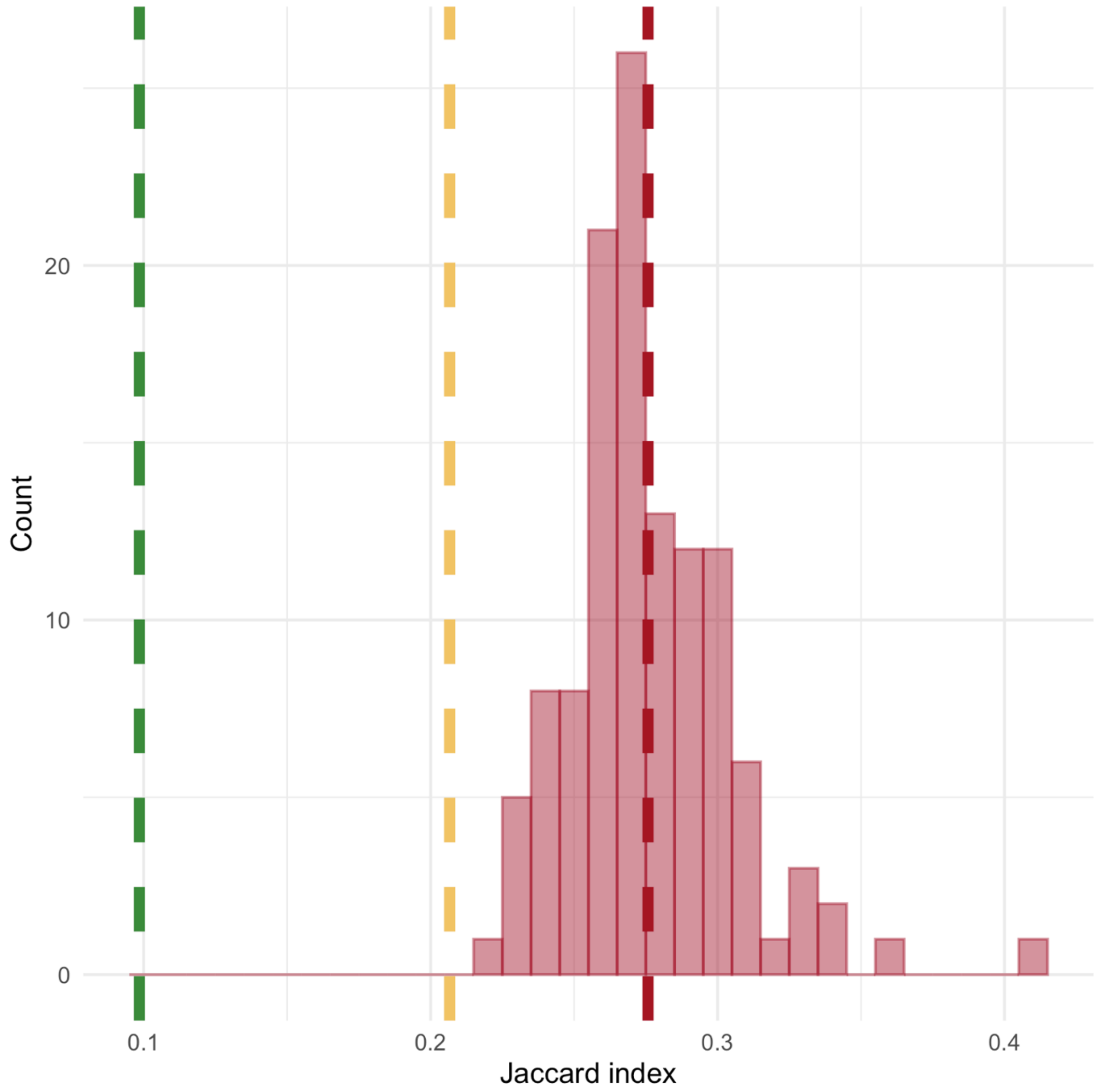

Supplementary Fig 4. Jaccard Index on sets of differentially expressed genes from all possible comparisons of 3 subsampled replicates from the within population data, where all possible combination of 3 replicates out of the 10 make up the permutations. The dashed red line represents the mean of the distribution, while the yellow is the mean value for the between population data and the green is the value for the between species data. Both means of between species and populations are significantly different from the permuted distribution of the within population data ($p = 0$).

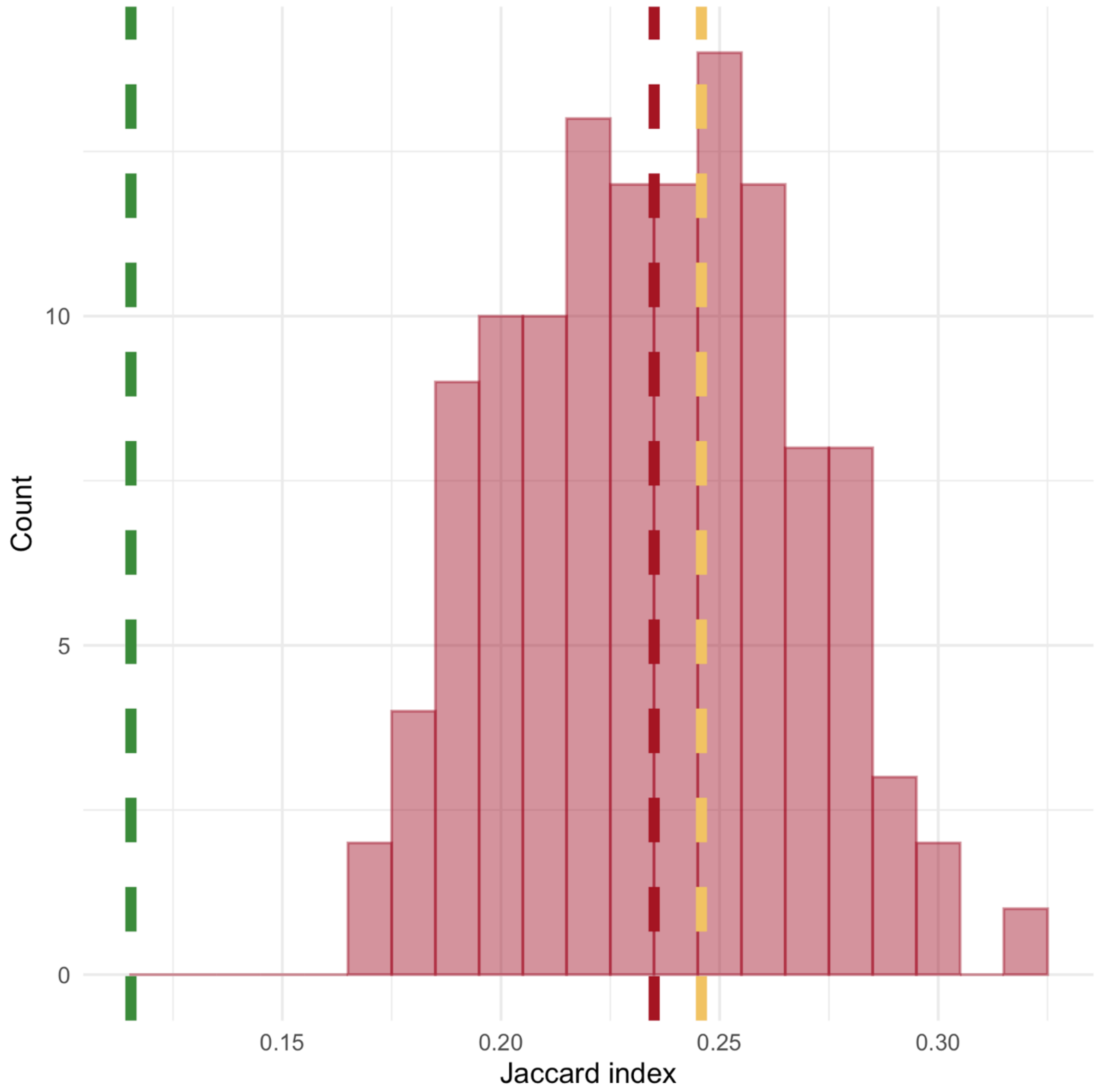

Supplementary Fig 5. Jaccard Index on sets of significantly enriched gene ontology terms from all possible comparisons of 3 subsampled replicates from the within population data, where all possible combination of 3 replicates out of the 10 make up the permutations. The dashed purple line represents the mean of the distribution, while the yellow is the mean value for the between population data and the green is the value for the between species data. The mean of between species was significantly different from the permuted distribution of the within population data ($p = 0$), but not the between population mean ($p = 0.6$).

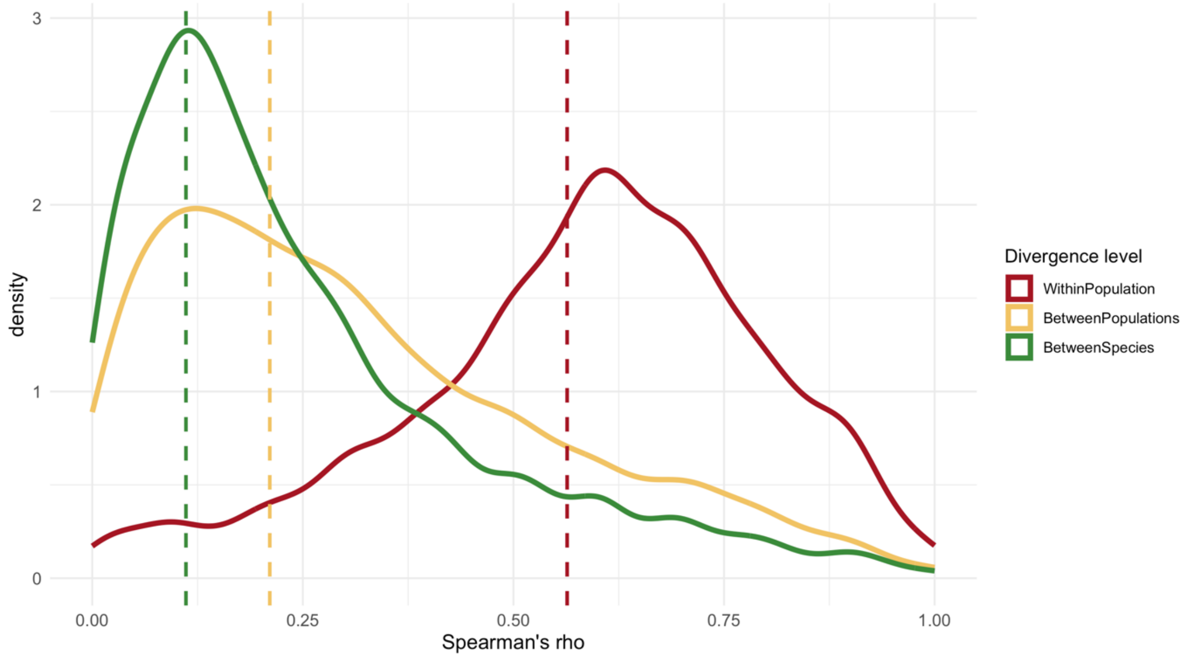

Supplementary Fig 6. Characterization of parallelism across gene ontology terms. Distributions of Spearman's *rho* across all genes in a given GO term in each divergence level. The dashed lines represent the mean of each distribution. In the case of the between populations and within population, where there are more than one pairwise comparison and therefore multiple correlation coefficients per GO term, one random correlation coefficient from a single pairwise comparison was used.
